# Virus Engineering and Applications

**DOI:** 10.3390/ijms242316788

**Published:** 2023-11-27

**Authors:** Laura Menotti

**Affiliations:** Department of Pharmacy and Biotechnology, University of Bologna, 40126 Bologna, Italy; laura.menotti@unibo.it; Tel.: +39-051-2094731

This Special Issue highlights multiple facets of virus engineering, ranging from the dissection of the biological properties of individual viral functions in the context of safe genomic backbones, virus genetic modification for applications in gene therapy, oncolytic virotherapy and vaccine production, to the hurdles presented by quality control and the delivery of viruses for their final applications and finally to the simulation, prediction and validation of virus evolution.

SARS-CoV-2 handling and modification is a hot topic in virus engineering. This previously presented a matter of debate due to the risks posed by the manipulation of a pandemic virus and by the misuse of a technology described in too much detail [1]. In this SI, Cordsmeier et al. (contribution 1) present a reporter system to determine the properties of individual variants of SARS-CoV-2 Spike proteins, as a further development of their passage-free SARS-CoV-2 genome backbone cloned into a BAC (Bacterial Artificial Chromosome). They engineered a fluorescent marker in place of a deleted gene (ORF6) for both safety reasons and imaging purposes. They propose their system as a flexible platform with which different Spike variants (including variants of concern and local variants) can be easily probed to assess their impact on infectivity and potential as emerging variants. They highlight the future prospects of assaying their recombinants for transmissibility, pathogenicity and immune escape ability.

Virus emergence and evolution is a relevant theme, and for RNA viruses, it is entangled with their ability to exist and behave as a quasispecies. During the pandemic, SARS-CoV-2 demonstrated quasispecies behavior as well [2]. In this SI, Gregori et al. (contribution 2) tackle quasispecies evolution from a predictive and computational point of view, and test their method against an in-host quasispecies evolution dataset derived from a HEV infection clinically treated with Ribavirin, which has a mutagenic effect on the virus genome. They propose that their tool be translated for the majority of infections caused by RNA viruses. In this scenario, quasispecies bioinformatic analysis is a powerful tool with which to perform a precise follow-up of engineered RNA viruses.

Li et al. (contribution 3) present an extensive review on viral-vector-based gene therapy, providing the historical background and an overview of the major virus platforms used and the challenges they face according to their specific properties. They also highlight the advantages of virus-based interventions when combined with editing technologies like CRISPR/Cas, even if the latter are not yet fully developed or safe for routine clinical use e [3]. A caveat applies in particular to virus-based therapies that are intended to have a long-lasting effect, as adverse effects can also be delayed over a long period of time.

In the framework of virus-based gene therapy, two research papers in this SI address the issue of AAV small cargo capacity and vector purity assessment. One approach to overcoming the small size of AAV is to engineer AAV with genomes optimized for size and that incorporate adenine base editors (ABEs) [4]. Furthermore, Ferreira et al. (contribution 4) devised a strategy involving protein trans-splicing in a dual AAV-vector system, relying on high-efficiency inteins. Interestingly, they found that high-quality vector preparation plays a central role in increasing trans-splicing rates. This observation is directly related to the work of Aebisher et al. (contribution 5), who compare the pros and cons of analytical electrophoresis and chromatography methods to rapidly characterize AAV VPs of different serotypes. The authors face a conundrum. On the one hand, highly generic techniques like CE-SDS are compatible with a regulated quality control (QC) environment, but they do not allow for distinguishing between serotypes. On the other hand, technical platforms like RPLC and HILIC are compatible with mass spectrometry (MS) and allow for discriminating between variants derived from different post-translational modifications of the capsid proteins, but still lack compatibility with QC. 

In the field of virus engineering for vaccine development, the H9N2 threat [5] has been a subject of study. An et al. (contribution 6) generated a recombinant avian flu H9N2 vaccine strain in order to restore high titer production and to remove mammalian pathogenicity. To this end, they replaced the PB2 segment of the PR8 strain with the corresponding triple-mutated genomic segment of an H9N2 vaccine strain to prompt coordination with PB1 and PA in polymerase trimer formation, and used the appropriate HA and NA assortment for receptor affinity and virus tropism. With regard to retroviruses, Ortiz et al. (contribution 7) devised a novel vaccine strategy based on FeLV-Gag virus-like particles, exposing a portion of the FeLV fusogenic protein. The vaccine was administered either as DNA or a purified VLP vaccine. An immune response was elicited not against the fusogenic protein antigen but the capsid protein; therefore, the vaccine platform is amenable for the testing of more complex immunogens. This report is in line with the molecular approaches to veterinary vaccination established years ago [6].

Finally, the work by Reale et al. (contribution 8) reports on an approach for the systemic delivery of herpes simplex viruses engineered for oncolytic virotherapy for the treatment of metastases and deeply rooted tumors. They show that human monocytes can be loaded with oHSV and deliver via intravenous injection the oncolytic virus to tumors xenografted on a chicken egg chorioallantoic membrane. Since an off-target delivery by monocytes to non-tumor tissue cannot be excluded, further safety measures to be combined with the delivery strategy include specific oHSV engineering for full retargeting and tumor-specific replication and virolysis [7,8].
